# Machine learning-based identification of an oxidative phosphorylation signature for prognosis, immune infiltration, and drug sensitivity in ovarian cancer

**DOI:** 10.3389/fimmu.2026.1724930

**Published:** 2026-05-29

**Authors:** Luyao Kang, Zuchen Yang, Yanna Ding, Ying Wu, Caixia Ma, Yaping Wang, Canyu Li, Bilan Li, Gaili Ji

**Affiliations:** 1 Department of Gynecology and Obstetrics, Clinical and Research Translation Center, The Third Affiliated Hospital of Zhengzhou University, Zhengzhou, Henan, China; 2 Shanghai Key Laboratory of Maternal Fetal Medicine, Shanghai Institute of Maternal-Fetal Medicine and Gynecologic Oncology, Shanghai First Maternity and Infant Hospital, School of Medicine, Tongji University, Shanghai, China; 3 Department of Cardiology, The First Affiliated Hospital of Henan University of Science and Technology, Luoyang, Henan, China; 4 Department of Gynecology and Obstetrics, The Affiliated Central Hospital of Zhengzhou University, Zhengzhou, Henan, China

**Keywords:** KIF1A, machine learning, ovarian cancer, oxidative phosphorylation, prognosis

## Abstract

**Background:**

Ovarian cancer (OC) is a highly heterogeneous disease, and its metabolic characteristics also exhibit heterogeneity. However, the specific metabolic pathways that play a critical role in OC metabolism remain unclear. Additionally, the significance of genes related to the metabolic pathways in the prognosis and therapeutic outcomes has not been clearly defined.

**Methods:**

In this study, we utilized the Cancer Genome Atlas Program (TCGA), Genotype-Tissue Expression (GTEx), and multiple Gene Expression Omnibus (GEO) datasets to perform gene set enrichment analysis (GSEA) on 84 metabolic pathways from the Kyoto Encyclopedia of Genes and Genomes (KEGG). Through robust rank aggregation (RRA) analysis, we identified the most significantly altered metabolic pathways. By constructing the most robust machine learning model using genes related to the most significantly altered metabolic pathways and combining it with single-cell sequencing analysis results, kinesin family member 1A (KIF1A) was selected as the gene for subsequent biological level studies.

**Results:**

We identified oxidative phosphorylation (OXPHOS) as one of the core metabolic pathways in OC. The OXPHOS-related gene signature (OPRGS) was built using the random survival forest (RSF) and supervised principal components (SuperPC) methods, which emerges as a comparatively reliable risk factor for OC. Patients with high-risk scores exhibited higher ESTIMATE stromal-related scores, a significant positive correlation with tumor-associated fibroblasts, higher tumor immune dysfunction and exclusion scores, and lower programmed cell death protein-1 (PD-1) and cytotoxic T lymphocyte-associated antigen-4 (CTLA-4) immunophenoscores in the TCGA cohort, suggesting an immunosuppressive tumor microenvironment (TME) based on bioinformatic predictions. Additionally, higher OPRGS was associated with lower cancer stemness indices, resistance to paclitaxel but sensitivity to carboplatin, revealing complex biological behaviors of the tumor. Further analysis showed that high OPRGS were also correlated with high scores in cancer-related hallmark signaling pathways, such as Notch, angiogenesis, and epithelial-mesenchymal transition signaling pathways. By integrating single-cell RNA sequencing data, we identified KIF1A as a key gene for further investigation. Our findings indicated that KIF1A was upregulated in OC cell lines and might promote cell proliferation, invasion, and migration.

**Conclusion:**

This study constructed a new OPRGS for OC. It may serve as a potential indicator for predicting prognosis, immune infiltration, and chemotherapy drug sensitivity in OC patients.

## 1 Introduction

According to recent epidemiological data, ovarian cancer (OC) accounts for approximately 314,000 new diagnoses and 207,000 deaths annually worldwide, positioning it as the second most lethal gynecological malignancy following cervical cancer (1). OC is highly elusive and difficult to detect, with approximately 80% of patients diagnosed after the disease has spread to advanced stages, resulting in a five-year survival rate of less than 30% (2). While the standard treatment regimen, comprising the surgical debulking followed by platinum-based chemotherapy, which initially induces remission in most patients, the majority eventually experience disease recurrence, metastatic progression, and acquired resistance to chemotherapy. Therefore, a deeper exploration of the molecular mechanisms underlying OC development and progression, as well as the identification of new prognostic biomarkers and effective therapeutic targets, is crucial for improving clinical outcomes in patients.

Tumor metabolic reprogramming has emerged as a new hallmark of cancer (3, 4). The progression of OC is accompanied by aberrant activation of multiple metabolic pathways, including aerobic glycolysis, glutamine metabolism, and lipid metabolism. These metabolic rewiring provide critical support for rapid tumor cell proliferation, maintenance of redox homeostasis, immune evasion, and metastasis (5, 6). The metabolic phenotype of OC exhibits significant heterogeneity across different tumor subtypes (7, 8). For example, endometrioid OC shares features with both clear cell and serous subtypes. These tumors may rely on glutamine and display moderate glycolytic activity (9). In high-grade serous OC and OC stem cells, a high dependence on mitochondrial metabolism and enhanced antioxidant capacity have been observed (10–13). High levels of oxidative phosphorylation (OXPHOS) are frequently linked to resistance against DNA-damaging agents and chemotherapy drugs (14–16). The use of OXPHOS inhibitors such as rasidipine and esbitoxin increased the activity of reactive oxygen species within cells and reduced the survival rates of ID8 and OVCAR5 cell lines (17). Furthermore, a high-throughput study on OXPHOS confirmed that expression profiles of OXPHOS-related genes are correlated with patient prognosis in OC (18). Collectively, these findings underscore the importance of deeply understanding key metabolic pathways in OC and identifying molecular biomarkers with clinical predictive value to improve prognosis prediction and guide personalized treatment strategies.

Current research has established prognostic models for OC based on metabolic-related genes and OXPHOS pathway genes (18, 19). However, most existing studies independently model specific pathways, predominantly relying on Cox regression analysis. There is a lack of systematic research paradigms that first globally identify metabolic pathways before targeting key pathways to construct prognostic models. To address these limitations, Li et al. proposed a stable machine learning recursive feature elimination (StabML-RFE) strategy. This algorithm employs eight popular machine learning methods to select high-frequency features from the subset with the maximum stability value as robust biomarkers (20). It shows clear benefits in identifying biomarkers but is better suited for classification tasks than for predicting survival outcomes. Robust rank aggregation (RRA), a robust algorithm for integrating multi-dataset rankings, effectively identifies gene sets and signaling pathways consistently upregulated or downregulated across studies and has been widely applied in mining core driver pathways in cancer (21). While RRA effectively addresses the challenge of robust pathway identification across multiple datasets, it cannot independently perform prognostic modeling. Combining RRA with the method of Mime1 (22), which incorporates multiple algorithms, overcomes the limitations of single-algorithm approaches in model construction, thereby facilitating the selection of the optimal prognostic model.

**Figure 1:**
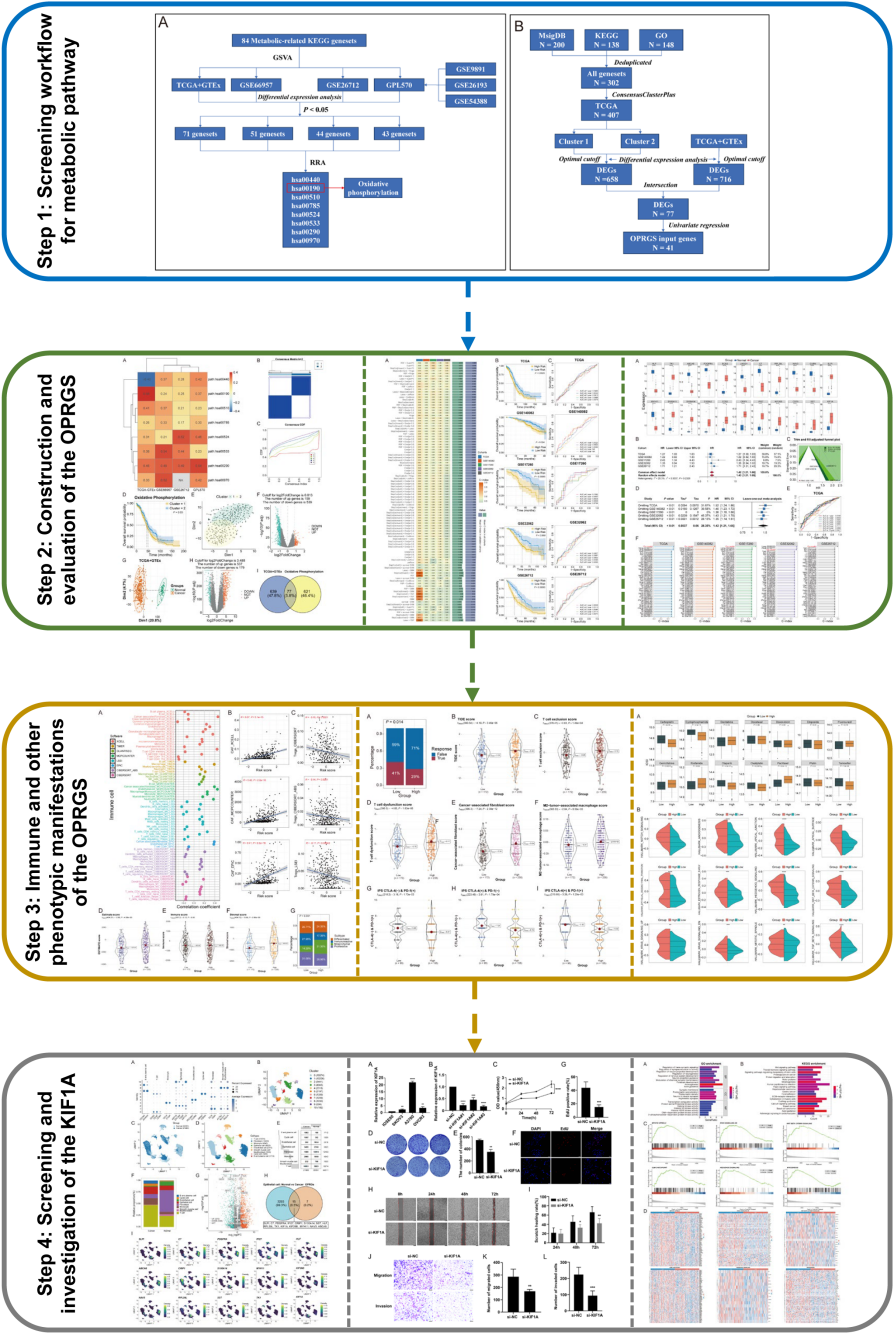
Flowchart of the study.

Based on this rationale, this study integrates transcriptomic datasets from multiple databases to unbiasedly screen core metabolic pathways in OC using the RRA algorithm. Subsequently, focusing on the identified OXPHOS pathway, we constructed and validated a novel OXPHOS-related gene signature (OPRGS) model based on the best-performing machine learning method. Additionally, we explored the relationships between OPRGS and tumor immune cell infiltration, immunotherapy, cancer stemness, and chemosensitivity. Finally, we verified the crucial role of the high-risk gene kinesin family member 1A (KIF1A) in regulating OC cell proliferation, migration, and invasion through *in vitro* experiments. These findings aim to provide new molecular evidence for prognostic stratification and personalized treatment in OC. The flowchart illustrating the study design was presented in **Figure 1**.

## 2 Materials and methods

### 2.1 Datasets sources

The Bulk RNA-seq data were obtained from Xena database (https://xenabrowser.net/datapages/) including the OC patients from the Cancer Genome Atlas Program (TCGA) dataset and normal ovarian patients from the Genotype-Tissue Expression (GTEx) dataset. OC patients categorized as type 01B, and with unknown expression levels and survival data were excluded for further analysis. Thus, there were 407 OC cases and 88 normal ovarian cases used in this study. The Gene Expression Omnibus (GEO) datasets (https://www.ncbi.nlm.nih.gov/geo/), including GSE9891 (n = 285), GSE26193 (n = 107), GSE54388 (n = 22), GSE26712 (n = 195), GSE66957 (n = 69) along with TCGA and GTEx data were used for screening of metabolic-related pathways. TCGA dataset was used for the construction of machine learning models. Four GEO datasets containing follow-up information, comprised of GSE32062 (n = 260), GSE17260 (n = 110), GSE140082 (n = 380) and GSE26712 (n = 185), were used for validation. For the TCGA and GTEx transcriptomic data, we employed count data for differential expression analysis (DEA), and the data was then subjected to the varianceStabilizingTransformation (VST) function from the R package “DESeq2” (v1.48.1) (23), which was used in other analytical processes beyond DEA. Utilizing the “removeBatchEffect” function within the R package “limma” (v3.64.3) (24), we integrated the datasets of GSE9891, GSE26193 and GSE54388 which were generated using the same sequencing platform (25). The survival time for all datasets is the overall survival (OS) time, with the survival outcomes categorized as either alive or deceased. All analysis scripts are available at https://github.com/Menz-Drina/OPRGS.

### 2.2 Screening and identification of metabolism-related pathway

Metabolic pathways were downloaded from the Kyoto Encyclopedia of Genes and Genomes (KEGG) database (https://www.genome.jp/kegg/) (26) using the package “KEGGREST” (v4.18.1). A total of 3056 human metabolic genes from the 84 metabolic pathways were listed in **Supplementary Table 1**. Gene set variation analysis (GSVA), a method of unsupervised gene set enrichment analysis, was employed to calculates the enrichment scores of predefined gene sets of each sample. In this study, GSVA was utilized to calculate the enrichment score of each KEGG metabolic pathway for each sample. Following GSVA analysis, we conducted differential analysis by R package “limma” to identify metabolically significant pathways with statistical significance (**Supplementary Tables 2**-**S5**).

**Figure 2:**
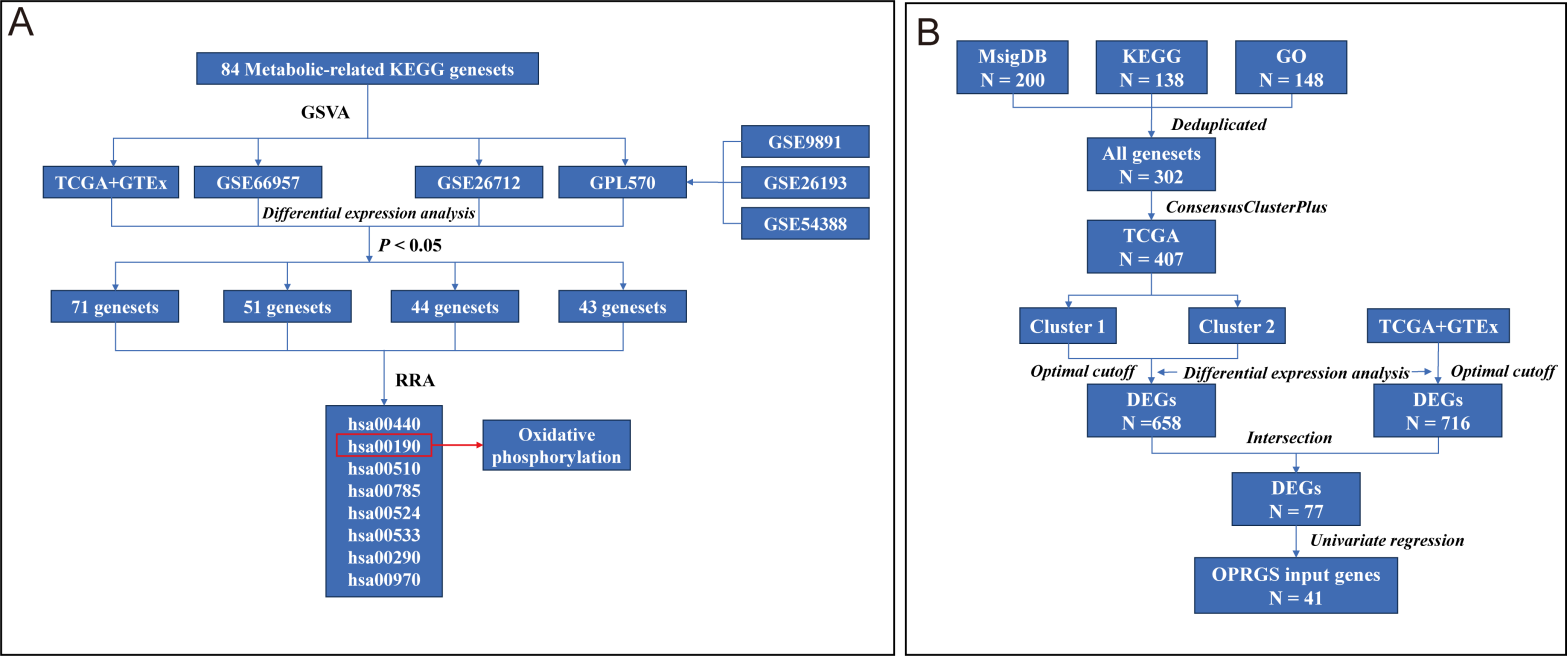
Screening flowchart of metabolic pathways **(A)** and OPRGS input genes **(B)**.

RRA analysis by “RobustRankAggreg” (v1.2.1) (21) package was used to integrate the enrichment score of each KEGG metabolic pathway with statistical significance (*P* < 0.05) from different datasets, including GPL570, GSE26712, GSE66957, TCGA and GTEx, in an unbiased manner using a comprehensive ranking list algorithm. The process of screening metabolic-related pathways was depicted in **Figure 2A**.

### 2.3 Identification the input genes of the OPRGS

Through RRA analysis, we selected the OXPHOS metabolic pathway with the best performance in the TCGA dataset for relevant analysis. We integrated genes related to OXPHOS from the KEGG (n = 138, **Supplementary Table 6**), Gene Ontology (GO) (n = 148, **Supplementary Table 7**) (27) and molecular signatures database (MSigDB) (n = 200, **Supplementary Table 8**) databases (https://www.gsea-msigdb.org/gsea/msigdb/index.jsp), obtaining 302 genes (**Supplementary Table 9**). Subsequently, we utilized the R package “ConsensusClusterPlus” (v1.72.0) (28) to perform consensus clustering analysis on the TCGA dataset with these genes. This analysis divided the TCGA dataset into two clusters with different survival outcomes using the R packages of “survminer” (v0.5.0) and “survival” (v3.8-3) and can be distinguished by t-distributed stochastic neighbour embedding (t-SNE) method using the R package “Rtsne” (v0.17). DEA was then carried out when the OC patients were divided into two clusters, specifically comparing the poor-prognosis cluster with the good-prognosis cluster.

We performed standard comparative mode of DEA on the count data of TCGA and GTEx using the R package DESeq2 for normal ovarian tissue versus OC tissue and for two subgroups of OXPHOS clustering, specifically comparing the poor-prognosis cluster with the good-prognosis cluster. The optimal cutoff for differential expression genes (DEGs) was set as mean(abs(log2FoldChange)) + 2 × sd(abs(log2FoldChange)), and the significance criterion for identifying DEGs was an adjusted *P*-value (False Discovery Rate, FDR) < 0.05. OPRGS input genes were selected through the intersection of DEGs and univariate regression with optimal cutoff point by the function of “surv_cox” from the R package “tinyarray” (v2.4.3). A total of 41 genes were used for subsequent OPRGS analysis (**Supplementary Table 10**). The **Figure 2B** illustrated the process of input genes screening for OPRGS analysis.

### 2.4 Construction and evaluation of the OPRGS

We used the package of “Mime1” (v0.0.0.90) (22) to calculate the Harrell’s concordance index (C-index) for the training dataset (TCGA dataset) and the validation datasets (GSE32062, GSE17260, GSE140082 and GSE26712). The prognostic model exhibiting the highest average C-index in the validation dataset was recognized as the most effective prognostic signature model for OPRGS. The risk score formula was calculated as follows: risk score = ∑ (coef × expression of genes).

Utilizing the R packages of “survminer” and “survival”, we stratified the OC patients into low OPRGS (low risk score) and high OPRGS (high risk score) groups. Using the “survival” package, we plotted the survival curves for both groups. The receiver operating characteristic (ROC) curve and time-dependent area under the curve (AUC) were used to quantify the discrimination performance and diagnostic value of the best OPRGS model of TCGA and GEO data in 1- to 5-year. Concurrently, we randomly collected 43 published predictive models on OC (**Supplementary Table 11**), employed the “Mime1” package to compute their C-indices to compare the predictive value with our model.

We used the hazard ratio (HR) for survival outcome to measure the prognostic value of OPRGS in OC by “Mime1” package (**Supplementary Table 12**). Heterogeneity across cohorts was estimated by using the I^2^ statistic. A fixed effects model was applied when the I² statistic exceeded 50%; otherwise, a random effects model was utilized. Publication bias was evaluated by the trim and fill method. Sensitivity analysis was conducted by leave-one-out meta-analysis to assess the influence of individual cohort on the overall result. Meta-analysis visualization, heterogeneity analysis, publication bias analysis and sensitivity analysis were performed by R package of “meta” (v8.2-0) (29).

### 2.5 Immune infiltration analysis in different OPRGS group

R packages of “IOBR” (v0.99.8) and “immunedeconv” (v2.1.0) were used to explore the correlation between risk score and immune cells. We then applied the function of “deconvo_tme” in IOBR to evaluate ESTIMATE score, which includes the estimate score, immune score, stromal score and Tumor purity score. The Tumor Immune Dysfunction and Exclusion (TIDE) score and the Immunophenoscore (IPS) were utilized to evaluate the effectiveness of OPRGS in predicting the therapeutic outcomes of immunotherapy in OC. The IPS of OC patients from the Cancer Immunome Atlas (TCIA) (https://tcia.at/home). The Immunotherapy response of OC patients was downloaded from TIDE (http://tide.dfci.harvard.edu/). We use the “ggbetweenstats” function from the “ggstatsplot” (v0.13.1) package for between-group comparisons.

### 2.6 Chemotherapy drug sensitivity and GSVA of hallmark gene sets in different OPRGS groups

Using R package “oncoPredict” (v1.2), we calculated the half-maximal inhibitory concentration (IC50) of 485 drugs in the TCGA dataset. We selected fourteen drugs of first-line and second-line for OC. Based on the OPRGS model, box plots of different drugs for TCGA dataset with two risk score groups were depicted using the wilcox.test method. Fifty hallmark gene sets were downloaded from MSigDB (h.all.v2025.1). The GSVA was performed to calculate the enrichment score of hallmark gene sets for each patient in the TCGA cohort.

### 2.7 Single-cell RNA sequencing (scRNA-seq) analysis

The scRNA-seq data including normal ovarian cases and OC cases was acquired from GEO database (GSE184880). R package “Seurat” (v4.3.0.1) (30) was used to integrate the samples. Quality control excluded low-quality cells using the following criteria: detected genes < 500 or > 8,000, mitochondrial gene proportion > 20%, ribosomal gene proportion < 3%, and hemoglobin gene proportion > 1%, which remained 44,011 cells. The resolution parameter in FindClusters of Seurat was used to determine the number of clustering groups. In this article, we chose the resolution of 0.1. Nonlinear dimensional reduction was performed using uniform manifold approximation and projection (UMAP) to visualize the scRNA-seq data. DEGs in scRNA-seq were identified using the FindMarkers function in Seurat. The criteria for DEGs of scRNA-seq were set as avg_log2FC of 2 and adjusted *P*-value < 0.05 (**Supplementary Table 13**). Cell clusters were defined based on the marker genes provided by Xu J et al. (31). Nebulosa was used to visualize the expression of genes in cells.

### 2.8 Enrichment analysis

The KEGG database (26), GO database (27) (https://geneontology.org/) and gene set enrichment analysis (GSEA) (32) (https://www.gsea-msigdb.org/gsea/index.jsp) were employed to conduct a comprehensive enrichment analysis of gene transcriptomic data using the R package “clusterProfiler” (version 4.12.0) (33), which facilitated the identification of the signaling pathways that the target genes may be involved in (34). We performed enrichment analysis using the “enrichGO” and “enrichKEGG” functions with *P*-value less than 0.05.

### 2.9 RNA extraction and real-time quantitative polymerase chain reaction (RT-qPCR)

Total cellular RNA was isolated using TRIZOL reagent (Cwbio, Jiangsu, China), and subsequently converted into complementary DNA. For RT-qPCR, SYBR Green PCR Master Mix (Toyobo, Osaka, Japan) was utilized. GAPDH was used as an endogenous control. The sequences of primers were as follows: KIF1A: sense: 5′-CACCACACCTCGTCAACCTGAAC-3′, antisense: 5′-GAACAATGTCCTGCCGCCTCTC-3′. β-actin: sense: 5′-CCTCGCCTTTGCCGATCC-3′, antisense: 5′-GGATCTTCATGAGGTAGTCAGTC-3′.

### 2.10 Transfection

Small interfering RNA (siRNA) KIF1A, as well as corresponding negative control siRNA (si-NC), were generated by GenePharma (Shanghai, China). For the cell transfection procedure, we used Entranster-R4000 (Beijing, China) to mediate transient transfection of cells. Transfection was allowed to incubate for 24 hours for RT-qPCR and 72 hours for western blotting. The sequences were as follows: si-KIF1A#1 sense: 5′-GCAGGACAUUGUUCUGAGUTT-3′, antisense: 5′-ACUCAGAACAAUGUCCUGCTT-3′; si-KIF1A#2 sense: 5′-GUCCUGCAGACAUCAACUATT-3′, antisense: 5′-UAGUUGAUGUCUGCAGGACTT-3′; si-KIF1A#3 sense: 5′-CAGCCGAUGAAGUCAACAATT-3′, antisense: 5′-UUGUUGACUUCAUCGGCUGTT-3′; si-NC sense: 5′-UUCUCCGAACGUGUCACGUTT-3′, antisense: 5′-ACGUGACACGUUCGGAGAATT-3′.

### 2.11 Cell Counting Kit-8 (CCK-8) assay

Cell viability was assessed utilizing the Cell Counting Kit-8 (SEVEN, Shanghai, China) following the instructions provided by the manufacturer. Cells were placed in 96-well microplates (Corning, New York, USA) at a density of 5,000 cells per well. Each well contained 100 μL of culture medium to support cell growth. At the intervals of 0, 24, 48, and 72 hours, 10 μL of CCK-8 reagent was meticulously added into each well. Subsequently, the plates were incubated for an additional two hours to facilitate the reaction. Absorbance measurements were then precisely recorded at 450 nm employing a microplate reader (BioTek, Vermont, USA) to assess cellular viability.

### 2.12 Colony formation assay

Cells were inoculated at a density of 1000 cells per well in six-well plates. Following an incubation period of 14 days at 37 °C, the samples were meticulously fixed with 4% Paraformaldehyde Fix Solution (Servicebio, Wuhan, China) for 15 minutes and subsequently stained with Crystal Violet Staining Solution (Servicebio, Wuhan, China) for 10 minutes with the staining time and crystal violet concentration adjusted according to the depth of cell staining. Colonies were counted using Image J.

### 2.13 Ethynyl-2′-deoxyuridine (EdU) staining

Cells were placed in 96-well microplates (Corning, New York, USA) at a density of 5 ×10^4^ cells per well. Each well contained 100 μL of culture medium to support cell growth. Cell viability was assessed utilizing the BeyoClick™ EdU-594 (Beyotime, Shanghai, China) following the instructions provided by the manufacturer.

### 2.14 Transwell assays

The Transwell assay was employed to assess the invasive and migratory capabilities of OC cells. For the invasion assay, the chamber was coated with Matrigel (BD Bioscience, USA) and a total of 1 × 10^5^ cells were cultured in serum-free medium and seeded into the upper chamber of Transwell inserts (Corning, New York, USA), whereas the lower chamber contained complete culture medium. Correspondingly, for the migration assay, a total of 8 × 10^4^ cells were cultured in serum-free medium and seeded into the upper chamber. Following a 48-hour incubation period, cell staining was conducted using the same protocol as that for the colony formation experiment. These samples were then examined using the IX71 inverted research microscope (Olympus, Shinjuku, Japan).

### 2.15 Scratch wound healing assay

The cells were meticulously seeded into a six-well plate and cultured until they attained approximately 90% confluency. Thereafter, a mechanical scratch was inflicted upon the cell monolayer utilizing a 200 µl pipette tip to simulate a wound. Floating cellsxwere removed through thorough washing with phosphate-buffered saline solution (SEVEN, Shanghai, China). Subsequently, the remaining viable cells were cultivated in complete medium. Morphological images were systematically captured at 0, 24, 48, and 72 hours. Image J was used to calculate the change in scratch area.

### 2.16 Statistical analysis

Statistical analyses and data visualization were conducted employing GraphPad and R software. Comparisons between two groups were carried out using t-test, while one-way ANOVA was applied for comparisons involving multiple groups. Chi-square test was utilized for the corresponding statistical analysis. A significance threshold of α = 0.05 was established for all tests. ^ns^*P* > 0.05, ^*^P < 0.05, ^**^P < 0.01, ^***^P < 0.001, ^****^P < 0.0001.

## 3 Results

### 3.1 Identification of the OXPHOS pathway

**Figure 3:**
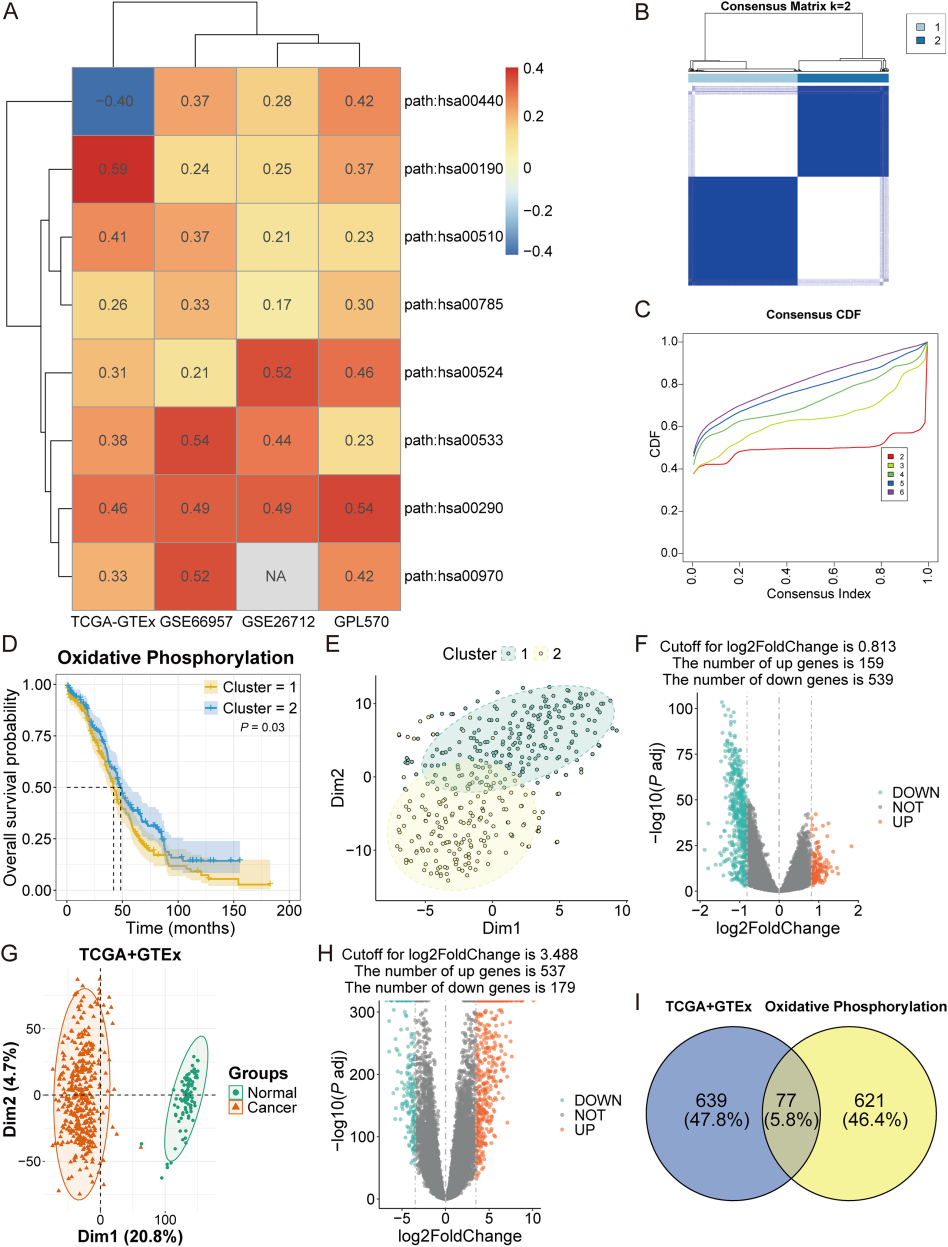
Identification of OXPHOS metabolic pathway and OPRGS input genes. **(A)** RRA result of GSVA followed by differential expression by limma. Consensus matrix heatmap **(B)**, CDF diagram **(C)**, survival analysis plot **(D)**, t-SNE **(E)**, and volcano plot of ConsensusClusterPlus clustering and DEA of OXPHOS genes **(F)**. PCA plot **(G)**, heatmap of and volcano plot **(H)** of differential expression genes between normal ovarian tissues (GTEx dataset) and OC tissues (TCGA dataset) **(H)**. Intersection analysis of OXPHOS genes and DEGs between OC tissues and normal tissues **(I)**.

We performed GSVA and differential analysis by R package of “limma” to identify metabolically significant pathways with statistical significance of 84 KEGG metabolic-related pathways for the TCGA and GTEx, GPL570, GSE26712, and GSE66957 (**Supplementary Tables 2**–**S5**), and selected statistically significant pathways for subsequent RRA analysis. During the RRA analysis, we generated heatmaps for the commonly significant pathways across the four datasets, revealing that the OXPHOS pathway exhibited the largest fold change in the TCGA dataset (**Figure 3A**). Therefore, we chose OXPHOS pathway for further analysis in this study.

### 3.2 Identification the input genes of OPRGS and their prognostic value

We downloaded relevant OXPHOS genes from the KEGG, GO and MSigDB databases, deduplicated the genes to obtain 302 OXPHOS genes. Consensus clustering analysis was conducted in TCGA dataset by R package “ConsensusClusterPlus”. The results indicated that dividing the OC patients into two clusters yielded clear and distinct color blocks in the heatmap of the consensus matrix (**Figure 3B**), and a distinct inflection point of the consensus cumulative distribution function (CDF) plot (**Figure 3C**). To assess the survival outcomes, utilizing the R packages “survminer” and “survival”, we performed Kaplan-Meier survival analysis to evaluate the time-to-event data. Results revealed a statistically significant disparity between the two clusters, with cluster 2 exhibiting a better prognosis (**Figure 3D**). Furthermore, using the R package “Rtsne”, t-SNE analysis confirmed clear separation between the two clusters (**Figure 3E**). And there were 698 DEGs selected (**Figure 3F**). As part of the quality control procedures prior to performing differential expression analysis, principal component analysis (PCA) (**Figure 3G**) was conducted by utilizing the R packages “FactoMineR” and “factoextra”. PCA analysis confirmed clear separation between OC and normal ovarian tissues (**Figure 3H**). DEA was conducted in the two groups with 716 DEGs. Following the intersection analysis with the results of DEA (**Figure 3I**), univariate regression was performed (**Supplementary Table 10**). Ultimately, we obtained 41 genes for constructing subsequent analyses.

### 3.3 Integrative machine learning algorithms to develop the OPRGS and evaluation its performance

**Figure 4:**
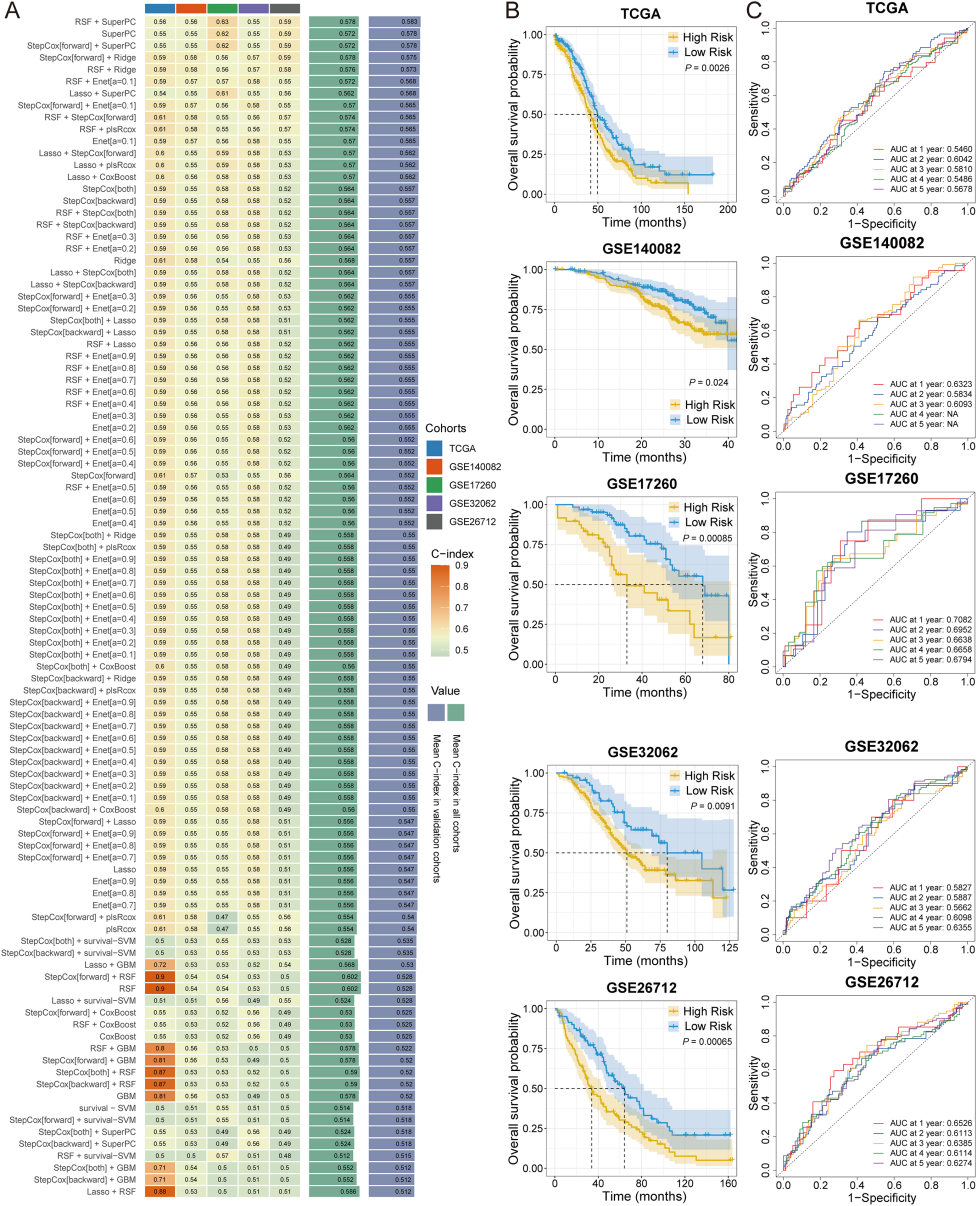
Identification of OPRGS using machine learning. **(A)** the C-index of 99 prognostic models developed with 10 different machine learning algorithms was evaluated in both training and validation cohorts. **(B)** the survival curves of OC patients stratified by OPRGS scores are presented. **(C)** the ROC curves for the predictive performance of OPRGS are shown across the TCGA, GSE140082, GSE17260, GSE32062, and GSE26712 cohorts.

We utilized TCGA dataset as the training set and four GEO datasets as the validation sets to construct a multi-model for OXPHOS for OC. The random survival forest (RSF) and supervised principal components (SuperPC) model achieved the highest C-index in all the validation cohorts (0.583) and maintained a high C-index in all cohorts (0.578) across all models (**Figure 4A**). The optimal OPRGS was developed by 22 gene-related features. The formula of the risk score was shown as follows: risk score = 0.5891×MYH11^exp^ + 0.7196×SLPI^exp^ + 1.0822×PDGFRA^exp^ + 0.4403×S100A2^exp^ + 1.4483×KIF26B^exp^ + (-0.9555)×RPL39L^exp^ + 0.3958×CRIP1^exp^ + (-0.4323)×CUBN^exp^ + 0.1271×S100A14^exp^ + 0.8469×CST6^exp^ + 0.8855×LMOD1^exp^ + 0.3078×KIF1A^exp^ + (-0.3404)×S100A11^exp^ + (-0.8485)×ECE2^exp^ + 0.3583×ABCA6^exp^ + (-1.3135)×SST^exp^ + 0.4882×NAV3^exp^ + 0.0385×HLF^exp^ + (-1.0155)×IFI27^exp^ + (-0.2759)×TK1^exp^ + (-0.7028)×CD52^exp^ + 0.7891×C7^exp^.

**Figure 5:**
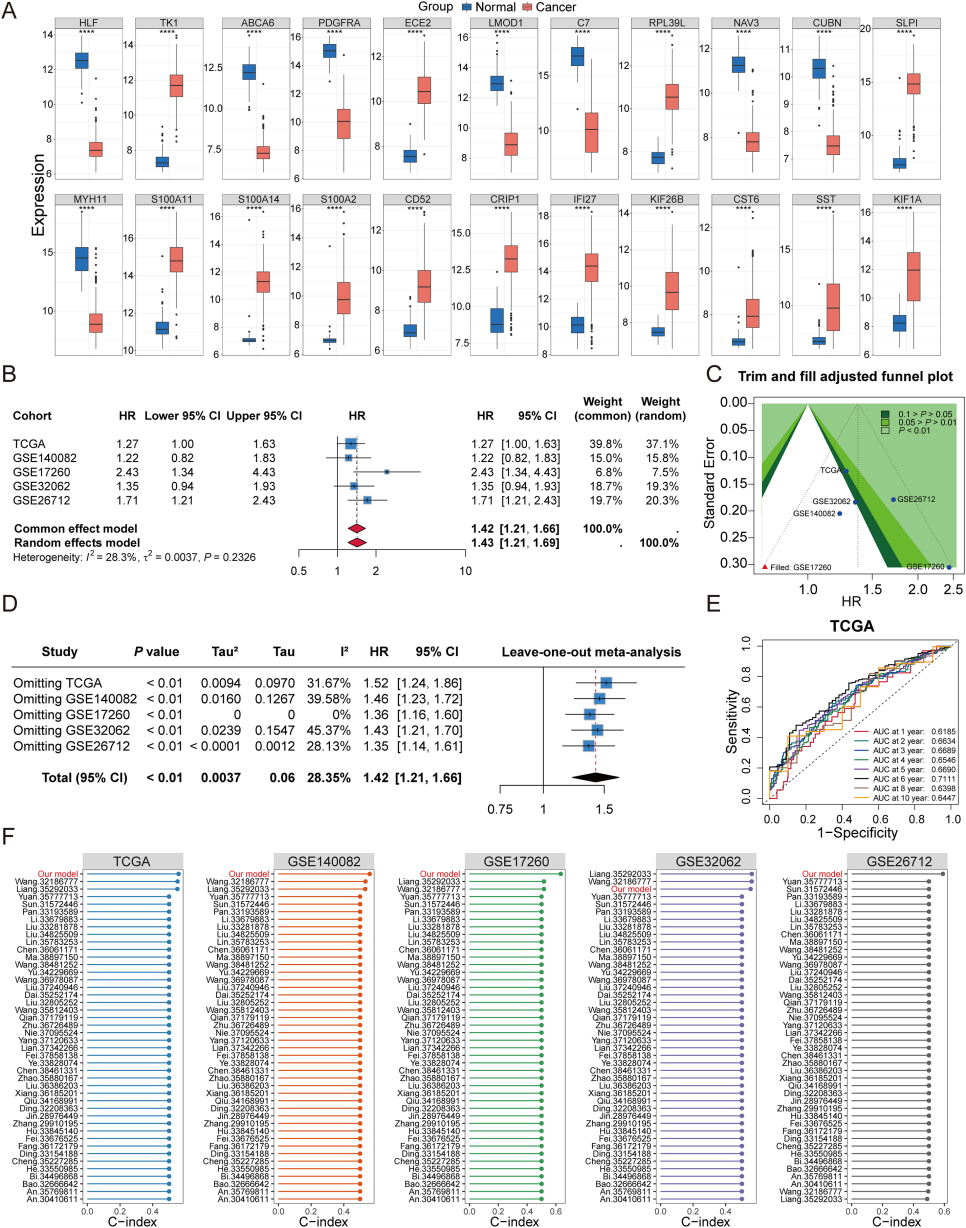
Assessment of the predictive capability of the OPRGS model for the prognosis of OC patients. **(A)** expression levels of 22 OPRGs identified by the RSF and SuperPC models in OC and normal ovarian tissues. **(B)** the meta-analysis was conducted to evaluate the prognostic significance of OPRGS. **(C)** the trim-and-fill analysis was used to evaluate the publication bias. **(D)** sensitivity analysis was conducted by leave-one-out meta-analysis. **(E)** ROC of the model combined OPRGS scores, age, tumor stage, tumor grade, and tumor size for OS at 1-, 2-, 3-, 4-, 5-, 6-, 8-, and 10-year. **(F)** comparison of the C-index between OPRGS and 43 other established prognostic signatures in forecasting outcomes for OC patients.

Subsequently, we constructed survival curves and ROC curves for both the training set and the four GEO test sets. The survival analysis revealed that patients with high OPRGS risk scores exhibited poorer prognoses, with statistically significant differences observed in both the training and validation sets (*P* < 0.05) (**Figure 4B**). Meanwhile, the ROC curve indicates that the OPRGS model exhibits relatively good diagnostic prognostic value (**Figure 4C**). The expression profiles of the 22 genes in normal and tumor tissues are depicted (**Figure 5A**). The results of the meta-analysis show that there was no significant heterogeneity among the five study groups (**Figure 5B**). The fixed-effects model reveals that the higher OPRGS scores were associated with poorer outcomes, which aligns with the survival analysis results. The trim-and-fill method revealed that the studies needing adjustment were located within the area of no statistical significance, suggesting possible publication bias (**Figure 5C**). The leave-one-out analysis confirmed that the meta-analysis findings possessed a certain degree of robustness (**Figure 5D**). We calculated the C-index for other prognostic indicators using the “coxph” function from the “survival” package. The C-index values for age (0.591), tumor stage (0.522), tumor grade (0.532), and tumor size (0.577) were obtained in individual analyses. When combined with our model in a multivariate setting, the C-index was moderately improved to 0.634. The time-dependent ROC curve, simultaneously combining OPRGS scores, age, tumor stage, tumor grade, and tumor size, demonstrated moderate predictive value (**Figure 5E**).

We also randomly selected 43 OC-associated prognostic signatures (**Supplementary Table 11**) and computed each C-index to evaluate the predictive performance of OPRGS relative to other prognostic signatures for OS in OC. The results indicated that the C-index of OPRGS ranked among the top three in all compared models (**Figure 5F**), suggesting the comparative prognostic predictive value of OPRGS relative to other models.

### 3.4 The immune microenvironment in OC patients with different OPRGS

**Figure 6:**
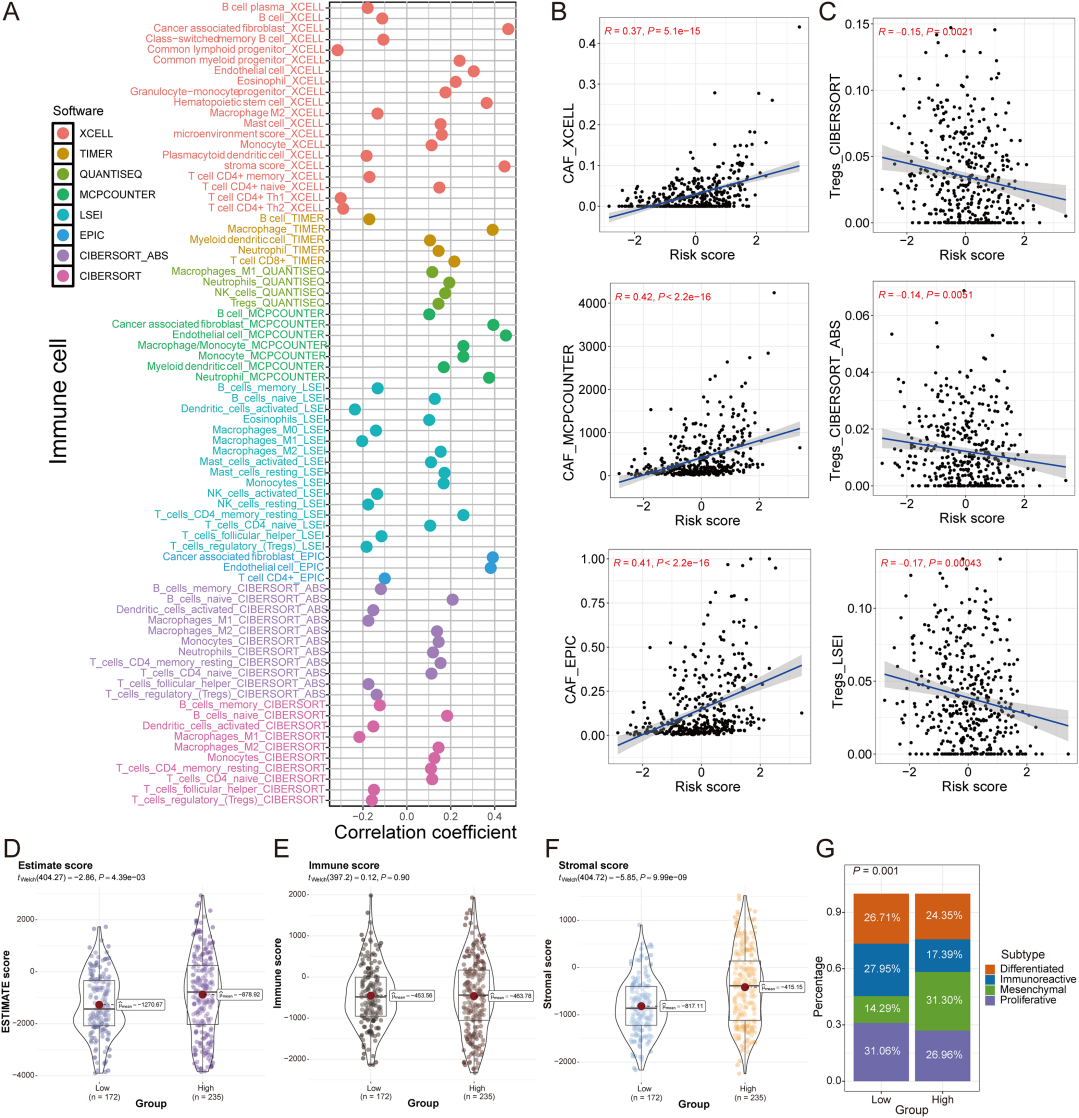
The relationship between the immune microenvironment and OPRGS in OC was investigated. **(A)** eight different computational methods were used to assess the association between OPRGS and immune cell infiltration in OC. The correlations between OPRGS and the levels of CAFs **(B)** and Tregs **(C)** were also examined. The ESTIMATE score **(D)**, immune score **(E)**, stromal score **(F)**, and subtypes of OC from TCGA **(G)** in different OPRGS groups.

OPRGS showed significant correlation with the abundance of immune cells or immune scores in TCGA dataset (all *P* < 0.05) (**Figure 6A**). Simultaneously, the infiltration of immune cells exhibits certain heterogeneity across different OPRGS groups. For instance, an elevated risk score was associated with an increased presence of cancer-associated fibroblasts (CAFs) (**Figure 6B**), while it corresponded to a decreased abundance of regulatory T cells (Tregs) (**Figure 6C**) in the TCGA dataset. This finding highlights the complexity and heterogeneity of the tumor microenvironment (TME). The immune infiltration of ESTIMATE analysis indicated that a higher risk score was associated with a greater stromal component within the tumor (**Figures 6D, F**), while the immune scores did not show statistically significant differences (**Figure 6E**). We also evaluated the composition of different subtypes of TCGA dataset across different OPRGS groups, which showed that there are significant differences in the distribution of subtypes by chi-square test (**Figure 6G**).

**Figure 7:**
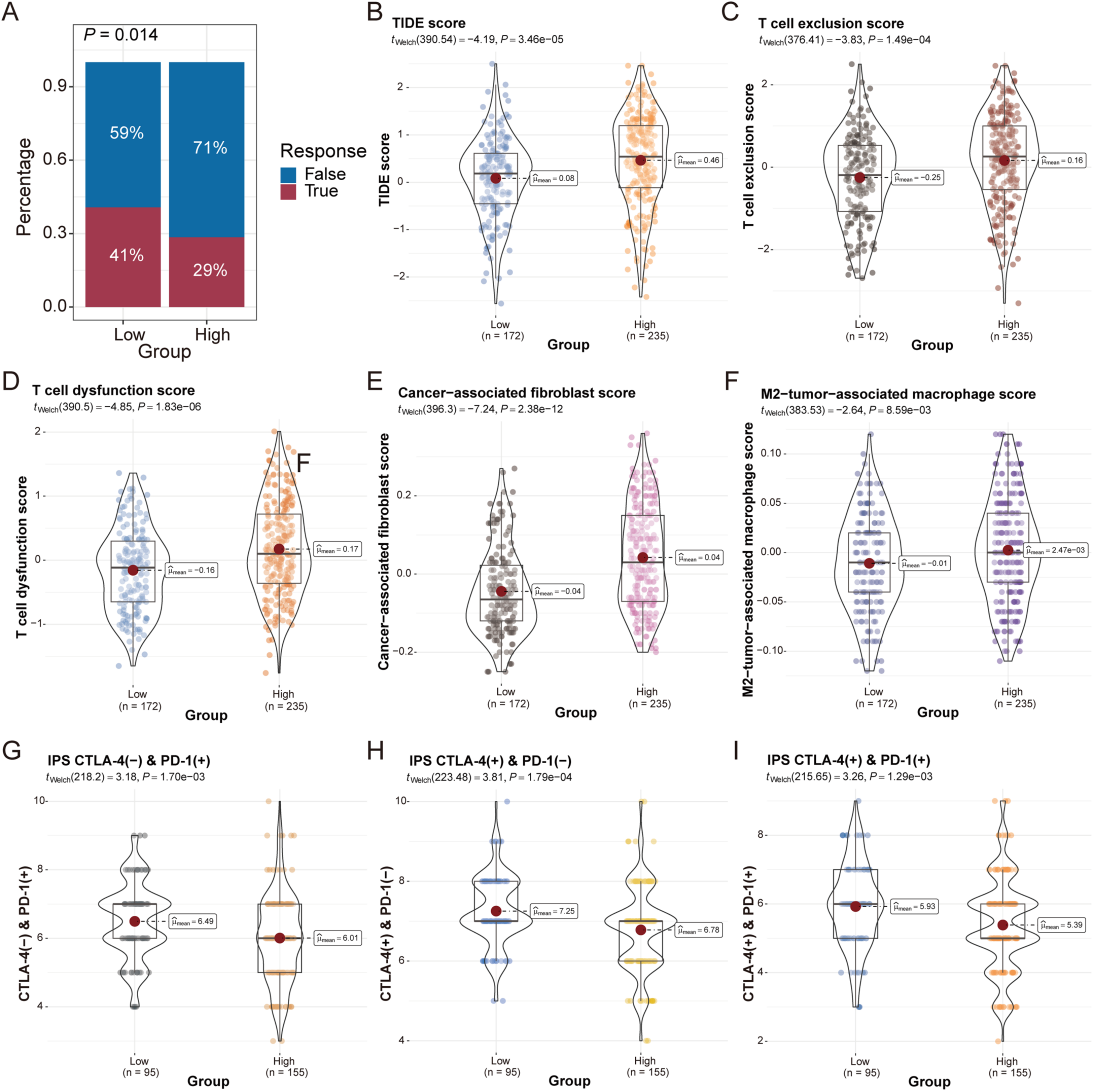
OPRGS as a biomarker for predicting immunotherapy outcomes in OC. Immunotherapy response **(A)**, TIDE score **(B)**, T cell exclusion **(C)** and dysfunction score **(D)**, cancer-associated fibroblasts score **(E)**, M2-tumor-associated macrophage score **(F)** and immunophenoscore **(G–I)** in different OPRGS score group.

Analysis using the TIDE database indicated that a higher proportion of non-responders to immunotherapy is observed in high-OPRGS group (**Figure 7A**), accompanied by elevated TIDE score (**Figure 7B**), T cell exclusion score (**Figure 7C**), T cell dysfunction score (**Figure 7D**), cancer-associated fibroblast score (**Figure 7E**), and M2-tumor-associated macrophage score (**Figure 7F**). Furthermore, the TCIA database was employed to assess the IPS scores of OC patients in different risk subgroups. The findings showed that the overall IPS, as well as the IPS for programmed cell death protein-1 (PD-1) and cytotoxic T lymphocyte-associated antigen-4 (CTLA-4), were significantly higher in the low-OPRGS group compared to the high-OPRGS group (**Figures 7G–I**).

### 3.5 Chemotherapy drug sensitivity and GSVA of hallmark gene sets in OC patients with different OPRGS

**Figure 8:**
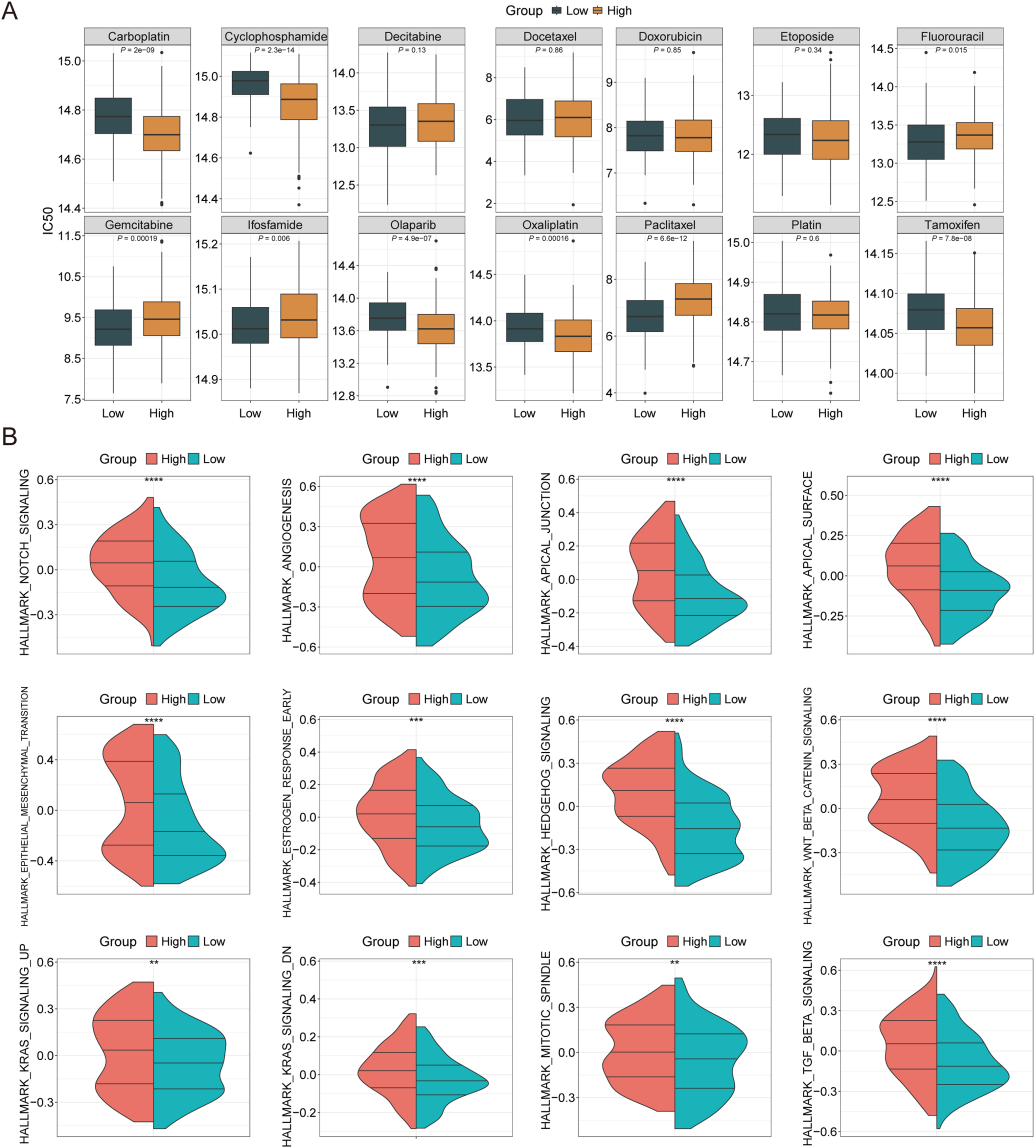
Chemotherapy drug sensitivity and GSVA of hallmark gene sets in OC patients with different OPRGS. **(A)** Chemotherapy drug sensitivity. **(B)** GSVA of hallmark gene sets. ^**^P < 0.01, ^***^P < 0.001, ^****^P < 0.0001.

Chemotherapy drug sensitivity revealed that the high-risk group exhibited increased sensitivity to carboplatin, oxaliplatin, cyclophosphamide, olaparib, and tamoxifen; decreased sensitivity to paclitaxel, fluorouracil, gemcitabine, and ifosfamide (**Figure 8A**). We ultimately conducted the GSVA to investigate the underlying mechanisms driving the progression of OC. An increased risk score was associated with an elevated level of Wnt/β-Catenin signaling activity, TGF-β signaling, NOTCH signaling, mitotic spindle signaling, KRAS signaling, Hedgehog signaling, early estrogen response signaling, epithelial mesenchymal transition signaling and apical junction signaling in TCGA (**Figure 8B**; all *P* < 0.05).

### 3.6 scRNA-seq analysis of OC patients

**Figure 9:**
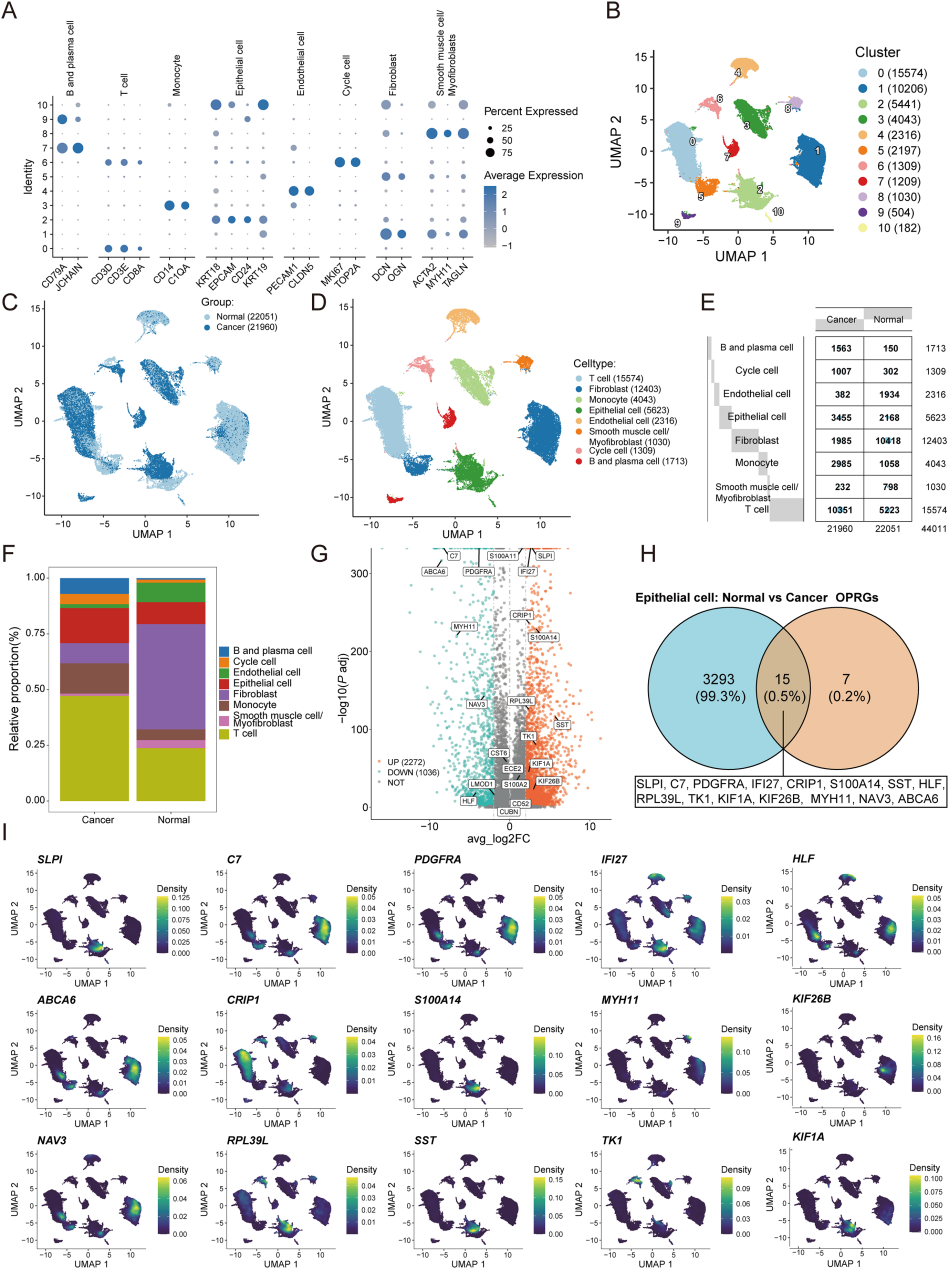
The expression patterns of OPRGs in scRNA-seq. **(A)** dotplot illustrating the average expression levels of cellular markers. **(B)** identified eleven cell types of from twelve ovarian cases of GSE184880. UMAP plots of overall distribution of cells from the tumor and normal groups **(C)** and distinct clusters of single-cell samples identified by cell clustering **(D)**. balloon plot **(E)** and histogram plot **(F)** of the proportion of different single-cell subpopulations in tumor and normal groups. DEGs in OC tissues compared to normal ovarian tissues within epithelial cell subpopulations **(G)**. intersection analysis of OPRGs and DEGs between OC tissues and normal tissues within epithelial cell subpopulations **(H)**. distribution of 15 intersected genes across various single-cell subpopulations **(I)**.

We divided GSE184880 OC patients into 8 main cell clusters (**Figures 9A–F**). The volcano plot illustrated the DEGs within a population of epithelial cells (**Figure 9G**) with 2272 upregulated genes and 1036 downregulated genes (**Supplementary Table 13**). Notably, 22 OPRGs were highlighted in the volcano plot and 15 OPRGs were identified as DEGs (**Figure 9H**). The expression of these genes in the cell clusters was shown in **Figure 9I**. Among these genes, SLPI, S100A14, RPL39L, SST, KIF1A, KIF26B, and MYH11 exhibit cell-specific distribution patterns. Specifically, SLPI, S100A14, RPL39L, SST, and KIF1A are predominantly expressed in epithelial cells. KIF26B is mainly distributed in fibroblasts, while MYH11 is mainly present in smooth muscle cells. In contrast, the remaining genes show a relatively broad distribution pattern: among them, C7, PDGFRA, HLF, ABCA6, and NAV3 are mainly present in T cells, fibroblasts, and epithelial cells; while IFI27 has the widest distribution, being distributed in endothelial cells, smooth muscle cells, monocytes, epithelial cells, and fibroblasts. Combining scRNA-seq analysis, gene univariate and multivariate regression, survival analysis, and gene expression data, we finally selected KIF1A, which performed robustly in both univariate and multivariate regression, as the follow-up gene.

### 3.7 Knockdown of KIF1A inhibited the progression of OC *in vitro*

**Figure 10:**
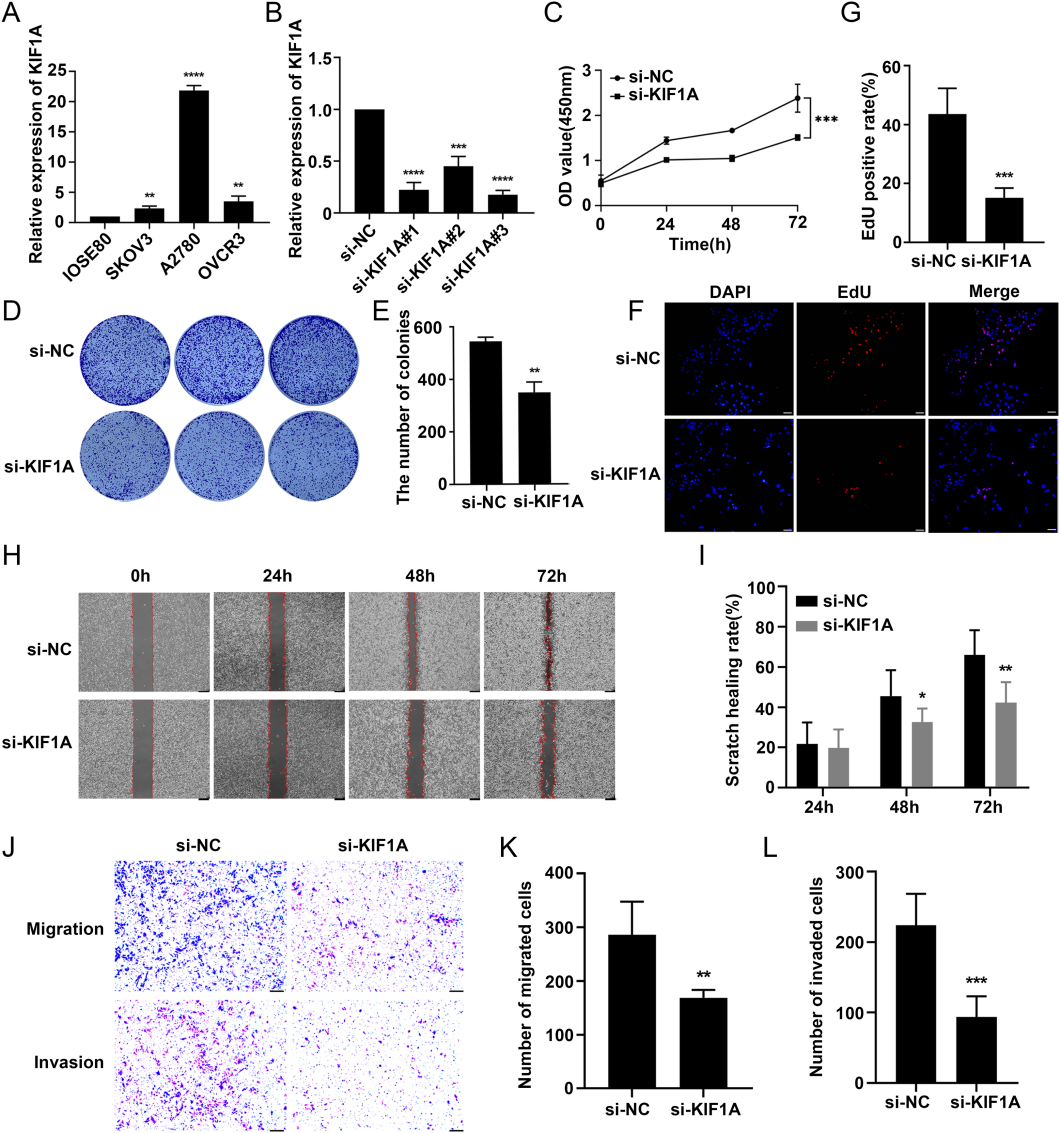
Knockdown of KIF1A inhibited the progression of OC *in vitro*. **(A)** expression levels of KIF1A in different OC cell lines. **(B)** RT-qPCR was used to verify the knockdown efficiency of different gene sequences of si-KIF1A in A2780 cell line. CCK-8 **(C)**, colony formation assay **(D, E)**, and EdU labeling assay (magnification, 200×) **(F, G)** were conducted to evaluate the proliferative capacity of OC cells transfected with si-KIF1A compared with those transfected with si-NC in A2780 cell line. Scratch wound healing assay (magnification, 40×) **(H, I)** and transwell migration assay (magnification, 100×) **(J, K)** and were conducted to evaluate the influence of KIF1A on the migratory capacity of A2780 cells. **(J, L)** Transwell invasion assay was conducted to evaluate the impact of KIF1A on the invasive capability of A2780 cells (magnification, 100×). The cell experiment was repeated three times. The data were expressed as mean ± standard deviation and analyzed by the t-test. ^*^P < 0.05, ^**^P < 0.01, ^***^P < 0.001.

Compared to normal ovarian cell lines (IOSE80), A2780 exhibited the highest mRNA expression level of KIF1A (**Figure 10A**). Then we selected A2780 for constructing a knockdown model *in vitro* to investigate the effects of KIF1A on cell proliferation, invasion, and migration for OC. Findings from RT-qPCR confirmed that the knockdown efficiency of si-KIF1A#3 was the highest (**Figure 10B**), which was used for further experiments. The alteration of proliferative ability after transfection of si-KIF1A#3 and si-NC for A2780 cells was then evaluated by CCK-8 assay (**Figure 10C**), colony formation assay (**Figures 10D, E**) and EdU labeling assay (**Figures 10F, G**). The results indicated that silencing of KIF1A markedly inhibited the proliferative ability of OC cells. To further assess the impact of KIF1A knockdown on the metastatic potential of A2780, we performed complementary invasion and migration assays. Transwell and scratch wound healing assays showed that knockdown of KIF1A dramatically restrained migration ability (**Figures 10H–K**) and invasion ability (**Figures 10J, L**) of the A2780 cell line.

### 3.8 Single-gene enrichment analysis results of KIF1A

**Figure 11:**
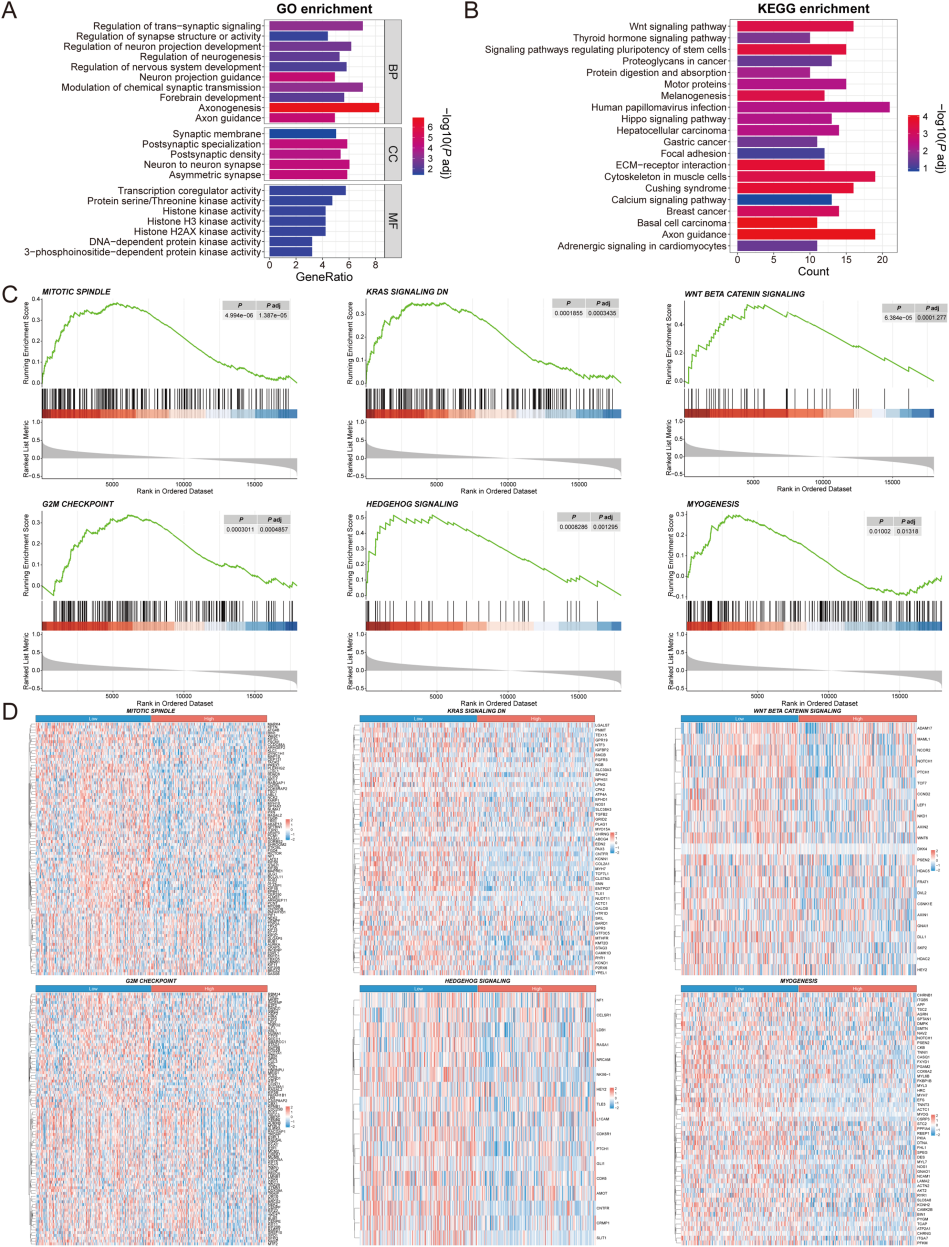
Single-gene enrichment analysis results of KIF1A. GO **(A)** and KEGG **(B)** enrichment analysis of KIF1A. GSEA of hallmark gene set for KIF1A. **(C)** the first six up-regulated pathways in GSEA enrichment analysis; **(D)** heatmap of the core genes in the first six pathways of GSEA enrichment analysis grouped by high and low KIF1A expression levels.

To investigate the potential involvement in signaling pathways of KIF1A, we analyzed transcriptome data from 407 OC patients obtained from TCGA. A total of 624 genes that exhibited a positive correlation with KIF1A were identified, characterized by a correlation coefficient exceeding 0.3 and a *P*-value less than 0.05. The findings from GO (**Figure 11A**) and KEGG (**Figure 11B**) enrichment analysis indicated that KIF1A was significantly associated with neuronal synaptic signaling, histone metabolism, Wnt, Hippo signaling pathways, and their correlation with the metabolism of various tumors.

To further investigate the signaling pathways potentially associated with KIF1A, we performed GSEA using the human hallmark gene sets from MSigDB. The analysis aimed to identify key pathways in which KIF1A might be involved. The GSEA results revealed that KIF1A was implicated in seven upregulated signaling pathways, mainly including the negatively regulated genes activated by KRAS, mitotic spindle activity, Wnt/β-Catenin pathway, the G2M checkpoint pathway and Hedgehog pathway (**Figure 11C**). Additionally, the heatmap of KIF1A expression-based stratification revealed that core genes within the upregulated pathways were significantly expressed in the high KIF1A expression group (**Figure 11D**). The GSEA results suggested that KIF1A was involved in various signaling processes closely related to tumorigenesis, implying that KIF1A may contribute to the development and progression of OC.

## 4 Discussion

Recent data has indicated that OXPHOS is the more likely form of energy metabolism in some cancer cells, including OC (35, 36). Among the histological subtypes of OC, bioenergetics profiles have been identified. High-grade serous OC and Clear cell OC show enhanced OXPHOS (8, 35–39). Here, we have evidence supporting the importance of OXPHOS in OC, which may be more prominent than glycolysis in OC.

In this study, while investigating the metabolism of OC, we also found that the OXPHOS pathway may play a significant role. Through multi-model screening, we successfully constructed OPRGS. The C-index of OPRGS alone as a prognostic factor indicated moderate predictive performance, which was comparable to traditional clinical and pathological factors such as age, tumor stage, grade, and size. Notably, when integrated into a multivariate model with these factors, the C-index was moderately improved, suggesting it may not function as a superior standalone indicator but could provide complementary prognostic information. Comparison with 43 published OC prognostic models showcased the superior prognostic value of our model. The model demonstrated significant survival significance in TCGA, GSE140082, GSE17260, GSE32062, and GSE26712. Meta-analysis also indicated that OPRGS is a reliable independent risk factor for OC patients. While these findings confirm the prognostic accuracy and reliability of OPRGS, its moderate predictive performance represents a key limitation. This indicates that OPRGS alone cannot achieve high-precision prognostic prediction, and highlights the necessity of combining it with other clinical or molecular markers to improve predictive accuracy.

The model also predicted the correlations with immune infiltration patterns, immune therapy responses, and chemosensitivity. We found that high-risk scores are correlated with immune-suppressive microenvironmental features, such as the enrichment of CAFs. CAFs have been widely reported to reshape the extracellular matrix by secreting various cytokines, inhibit the function of cytotoxic T cells, thereby promoting tumor immune evasion. This aligns with our observed low immune scores and high TIDE scores, which may explain why high-risk patients do not respond well to current immune checkpoint inhibitor treatments to some extent (40–42). However, our findings were not validated in an independent cohort of OC patients receiving immunotherapy, which limits the clinical applicability and translational potential of OPRGS in immunotherapy.

In terms of treatment, the OPRGS model demonstrates potential in predicting chemosensitivity. High-risk patients exhibit greater sensitivity to drugs such as carboplatin and oxaliplatin, which may be related to their active metabolic state and DNA replication process, making them more susceptible to DNA damage induced by platinum-based drugs. Conversely, reduced sensitivity to drugs like paclitaxel, fluorouracil, and gemcitabine suggests the presence of different resistance mechanisms among various subtypes. These findings may provide certain references for clinical drug selection and lay a preliminary foundation for the development of personalized chemotherapy regimens based on patients’ OPRGS risk scores in the future. Further analysis showed that high OPRGS was also correlated with high scores in cancer-related hallmarks signaling pathways, such as Notch, angiogenesis, and epithelial-mesenchymal transition signaling pathways.

Through scRNA-seq analysis, we validated at the single-cell level that the OPRGS risk gene is predominantly expressed in epithelial cells and excluded several OPRGS risk genes that might originate from other stromal cells within the tumor microenvironment, thereby enhancing the specificity of the target genes in subsequent functional studies. Subsequently, we selected KIF1A for functional validation.

As a member of the kinesin family, KIF1A plays a crucial role in intracellular cargo transport and mitosis (43). Previous studies have reported that KIF1A promotes cancer progression in neuroblastoma (44), and prostate cancer (45). KIF1A has also been suggested to be associated with poor prognosis at the level of bioinformatics in OC (46). However, there is no relevant report on its biological function. This study demonstrated the pro-proliferative, migratory, and invasive roles of KIF1A in OC, suggesting its potential as a therapeutic indicator.

However, this study also has some limitations. Although we used multiple independent cohorts for validation, all data were derived from retrospective studies, and their conclusions still need to be further confirmed in prospective clinical research. The relationship between the OPRGS model and immune therapy response is primarily based on bioinformatics predictions, such as IPS and TIDE. Due to the lack of an external validation cohort for OC immunotherapy, the clinical evidence from OC patient cohorts receiving immunotherapy was insufficient. Therefore, the strength of this evidence was relatively weak which needs more immunotherapy data for OPRGS regarding the effects of immunotherapy. Besides, as a risk gene in the OPRGS signature, no direct experimental evidence currently links KIF1A to OXPHOS, mitochondrial function, or metabolic reprogramming. The specific molecular mechanisms by which KIF1A regulates the OXPHOS pathway or affects tumor progression have not been fully elucidated and require further investigation. Furthermore, it is necessary to refine *in vitro* experiments and *in vivo* experiments to better characterize the signaling pathways involving KIF1A and to elucidate its role in the mitochondrial function, metabolic reprogramming, and cancer progression of OC. All these efforts will address existing gaps and enhance our understanding of KIF1A’s function in OC.

In summary, this study identified OXPHOS as a key metabolic pathway in OC and constructed the OPRGS model. The OPRGS exhibited moderate but reliable performance for predicting risk stratification and prognosis in OC patients. Our comprehensive analysis revealed the role of OPRGS in the immune microenvironment and chemosensitivity of OC, and identified KIF1A as a novel oncogene and a potential therapeutic target. KIF1A may serve as a potential oncogene and therapeutic target for OC in future investigations.

## Data Availability

The original contributions presented in the study are included in the article/**Supplementary Material**. Further inquiries can be directed to the corresponding authors.
